# Impact of impulsivity on the relationship of the brain structures with school performance

**DOI:** 10.1038/s41539-025-00344-z

**Published:** 2025-08-08

**Authors:** Youngwoo Bryan Yoon, Wi Hoon Jung

**Affiliations:** 1 Department of Psychiatry, Columbia University Irving Medical Center, New York, NY USA; 2 New York State Psychiatric Institute, New York, NY USA; 3 Department of Child and Adolescent Psychiatry, New York University School of Medicine, New York, NY USA; 4 Department of Psychology, Gachon University, Seongnam, South Korea

**Keywords:** Intelligence, Psychology, Psychology

## Abstract

While prior research has explored the neurobiological mechanisms underlying adolescent school performance, these mechanisms remain poorly understood in college students. Impulsivity has been highlighted as a key factor affecting academic success; however, its influence on the relationship between school performance and brain structure remains underexplored. In this study, we used a sample of college students to investigate which gray matter volume (GMV) in brain regions was associated with grade point average (GPA), and whether impulsivity mediates these relationships. Our findings revealed correlations between GMV in the caudate nucleus (CN) and cerebellum with GPA. Increased CN was correlated with poorer GPA through higher impulsivity, whereas higher cerebellum was associated with better GPA through lower impulsivity. These results indicate that CN and cerebellum play crucial roles in school performance and associated impulsivity. Various interventions targeting impulsivity, such as therapy, counseling, and medication, could improve educational outcomes by addressing the underlying neurobiological factors.

## Introduction

Grade Point Average (GPA) is commonly used as an indicator of school performance, and has been identified as a much more accurate predictor of college success than standardized test scores^1^. Research on the impact of GPA is crucial, as it sheds light on how school performance correlates with a student’s ability to meet the demands of both higher education and the professional world^2^. A student’s GPA during college significantly influences their future career development, including job prospects and opportunities for advanced studies, such as graduate or professional programs^3^. Understanding the factors that influence GPA could allow educators and policymakers to provide better support, helping students to achieve higher academic standards, and improving their career prospects and overall life outcomes. Additionally, analyzing GPA trends can uncover gaps in educational resources and support systems, thereby facilitating targeted interventions to promote academic success, personal growth, and address structural inequalities in education^4–7^.

Extensive research has been conducted on the relationship between brain structure and intelligence, with historical studies highlighting the role of the frontal and parietal cortices (i.e., the Parieto-Frontal Integration Theory)^8^, while recent studies have started to emphasize the involvement of other brain regions, such as the striatum (especially the caudate nucleus [CN])^9^ and cerebellum^10^. Previous studies have reported that the striatum and dopamine play a critical role in cognitive functions such as memory and decision-making^11,12^. Both the putamen and caudate, which are part of the striatum, have been linked to various cognitive functions. However, research suggests that the caudate, more than the putamen, is associated with a broader range of cognitive functions beyond behavior, including goal-directed actions and strategic planning^13^. Additionally, several studies indicate that the cerebellum contributes to higher-order cognitive processes such as working memory, language, and problem-solving, expanding its traditional role in motor control^10,14–16^. However, only a few studies have specifically examined the connection between brain structure and school performance^17–23^. Wang et al. found a positive correlation between the density of the left dorsolateral prefrontal cortex (DLPFC) and high school students’ school performance^17^. Moreover, they reported that the involvement of the left DLPFC in academic performance accounted for more than one-third of the effect of impulsivity on academic outcomes^18^. Similarly, Ivanovic et al. reported significant associations between gray matter volume (GMV), the thickness of the prefrontal cortex—particularly the right inferior frontal gyrus—and scholastic achievement among high school graduates^19^. One longitudinal study involving children and adolescents reported that higher vocabulary scores were linked to the development of the left fusiform gyrus, and arithmetic scores were associated with the left striatum, indicating the potential involvement of motivational factors^21^. Other studies have reported the impact of sleep habits^22^ and children’s weight^20^ on academic performance and brain structure. These neuroimaging studies underscore the complex interplay between the brain structure and academic success, highlighting potential pathways for enhancing educational outcomes through a deeper understanding of the neural basis of school performance.

While these studies focused on children and adolescents and measured school performance using standardized tests, there is currently a notable gap in research exploring the relationship between brain structure and school performance, as assessed using GPA, in young adults, particularly college undergraduates. Filling this gap is critical because college represents a unique neurodevelopmental period characterized by continued maturation of brain regions involved in executive functions and impulse control, such as the prefrontal cortex (PFC), cerebellum, and striatum^24–28^. During this period, the PFC and its connections with subcortical regions (such as striatum) continue to develop, while individuals face unprecedented autonomy in academic decision-making^29,30^. Unlike younger students, college undergraduates must independently manage competing priorities, develop strategic approaches to diverse courses, and maintain focus despite reduced external structure^31^. College GPA represents the average performance across courses that college students have deliberately selected, encompassing strategic course choices, consistent class participation, thorough exam preparation, and engagement in academic activities^32^. These challenges make college an especially important period for understanding how brain structure relates to academic achievement and impulsivity^33,34^. Furthermore, college GPA encompasses more complex cognitive and behavioral processes than standardized tests, as it represents performance across multiple domains over extended periods with varying levels of supervision.

Psychological factors significantly influence school performance, shaping students’ academic success and overall educational experience^32,35^. Key factors include motivation, self-efficacy, and impulsivity. Motivation drives students to engage in their studies and persist through challenges^36^, while a strong sense of self-efficacy (defined as the belief in one’s own ability to succeed) can enhance school performance by boosting effort and resilience^31,37^. In contrast, high impulsivity can hinder cognitive functioning and concentration, leading to reduced academic achievement^38^. Psychological well-being, particularly emotional regulation related to impulsivity, plays a crucial role in students’ abilities to manage academic demands and maintain focus^34,39,40^. Impulsivity, as a multidimensional construct encompassing attentional, motor, and non-planning tendencies^41,42^, is closely linked to cognitive processes such as executive functioning and decision-making^43,44^. These processes are critical for academic success, as they influence students’ ability to prioritize tasks, regulate emotions, and resist distractions^45,46^. Neurobiological evidence suggests that brain regions implicated in impulsivity, such as the caudate nucleus and cerebellum, also play roles in reward processing, habit formation, and cognitive control^47–49^. Thus, impulsivity may serve as a key mediator connecting structural brain differences to variations in academic outcomes like GPA. In addition, impulsivity has been identified as a key determinant of school performance, potentially leading to issues such as higher dropout rates and delinquency^50,51^, thus underscoring the importance of understanding how impulsivity impacts school performance. Insights into the role of impulsivity and its associated neural markers are crucial for developing effective educational strategies and support systems to improve academic outcomes and student well-being. However, only a few studies have examined how brain regions influence school performance, specifically in their role as mediators of impulsivity in college students.

We hypothesized that there would be close links among impulsivity, school performance, and the underlying neural mechanisms, specifically in the striatum and cerebellum, as highlighted in previous studies on intelligence and working memory^9,10^. Therefore, the present study aimed to explore (i) the association between brain structures (GMV estimated by the voxel-based morphometry [VBM] approach) and school performance (as indicated by GPA), and (ii) whether individual impulsiveness mediates the relationship between the above-identified brain structures and GPA, and whether the brain structures mediate the relationship between impulsivity and GPA. We used the Barratt Impulsiveness Scale version 11 (BIS-11), a comprehensive measure of trait impulsivity based on research on anxiety and sensation/thrill seeking, to assess individual impulsiveness. The study was conducted as follows. First, we performed a multiple linear regression analysis to determine the regions associated with GPA at the whole-brain voxel level. Second, we examined whether the brain structures identified above were linked to individual impulsivity. Finally, we explored whether impulsivity mediated the relationship between brain structures and GPA. A post-hoc analysis was also conducted to test whether brain structure mediated the relationship between impulsivity and GPA.

## Results

### Neural correlates of school performance

**Fig. 1:**
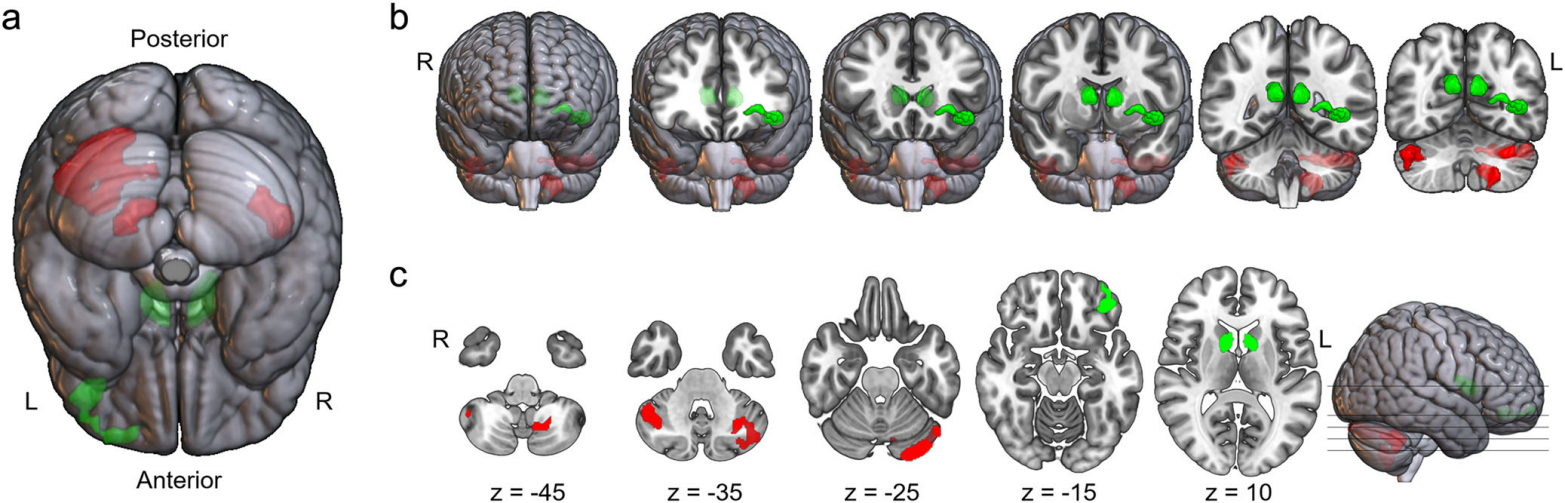
Brain regions associated with school performance. A total of six clusters were significantly associated with school performance in the whole-brain regression analysis, defined by an FWE-corrected *p* < 0.05 at cluster-level, combined with uncorrected *p* < 0.001 at the height level (refer to Table 2). **a**, **b** 3D rendering of the clusters associated with school performance. Red and green clusters indicate regions positively and negatively associated with school performance, respectively. **c** Axial slices showing clusters of brain regions associated with school performance. L left, R right.

Participant characteristics, including age, sex, handedness, education level, BIS-11 total score, and GPA, are summarized in Table 1. Table 2 provides the statistical results of the whole-brain regression analysis performed to detect the brain regions related to school performance (at a cluster-forming height threshold of *p* < 0.001 (uncorrected) and correcting for multiple comparisons with a cluster extent threshold of *p* < 0.05). The GMVs of three clusters in the cerebellum were positively associated with an individual’s level of school performance, whereas the GMVs of the left lateral orbitofrontal cortex and bilateral CN were negatively associated with an individual’s level of school performance (Fig. 1).

**Table 1:** Demographics of participants

Variable	Value
Total number of participants	153
Age (years)	22.57 ± 2.77
Sex (male/female)	76/77
Handedness (right-handed/left-handed)	148/5
Education (years)	15.11 ± 1.38
Total Score of BIS-11	48.96 ± 10.28
GPA (range: 2.30–4.50)	3.93 ± 0.48

**Table 2:** Brain regions showing significant correlations with school performance

Areas	MNI coordinates (*x*, *y*, *z*)	*t* value	*Z* value	Voxels
Positive association with school performance				
Cerebellum crus1	−22, −88, −24	4.56	4.40	2075
Cerebellum 8	−20, −64, −45	4.54	4.38	500
Cerebellum crus1	45, −54, −34	4.19	4.06	702
Negative association with school performance				
Lat. OFC	−42, 40, −14	5.42	5.16	546
Caudate nucleus	−15, 4, 8	4.54	4.38	509
Caudate nucleus	6, 6, 12	4.40	4.26	564

*Lat* Lateral, *OFC* Orbitofrontal Cortex.

### Association between the identified regions and impulsivity and between impulsivity and school performance

**Fig. 2:**
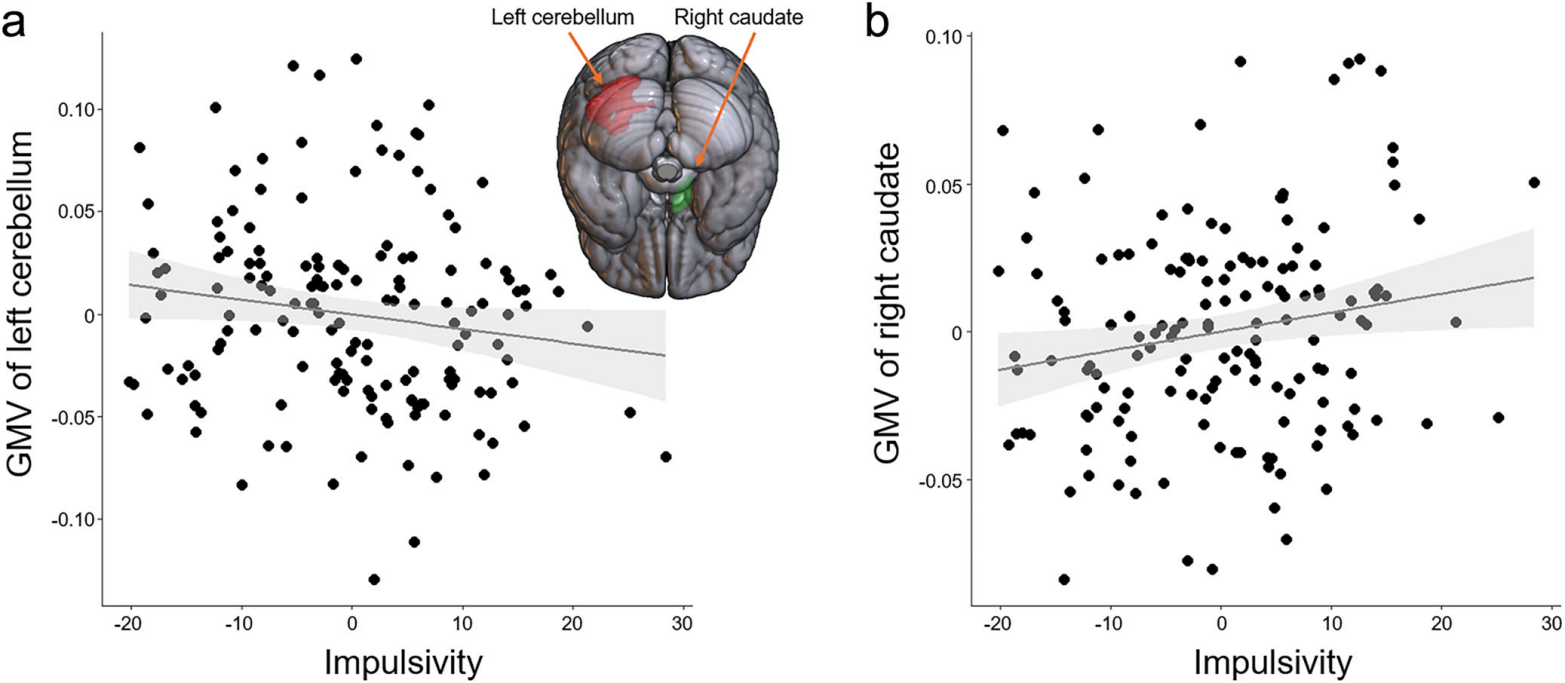
Associations between gray matter volumes of identified regions and impulsivity. Two of the regions identified from the whole-brain regression analyses showed a significant association with impulsivity, as measured by the total score of BIS-11. **a** The gray matter volume (GMV) of the left cerebellum showed a negative association with impulsivity. **b** The GMV of the right caudate nucleus showed a positive association with impulsivity. For illustration purposes, the scatterplots here were created, with a linear regression line added between residuals obtained after regressing out age, sex, handedness, duration of education, and total intracranial volume.

The Spearman correlation analyses showed that the GMVs of two identified six clusters (Table 2) showed a significant correlation with impulsivity (Fig. 2). Specifically, the GMV of the left cerebellum (MNI *x*, *y*, *z* coordinates = −22, −88, −24) was negatively correlated with impulsivity (total score of BIS-11; *r* = −0.170, *p* value = 0.036) (Fig. 2a), while the GMV of the right CN (MNI *x*, *y*, *z* coordinates = 6, 6, 12) was positively correlated with impulsivity (*r*-value = 0.164, *p* value = 0.042) (Fig. 2b).

**Fig. 3:**
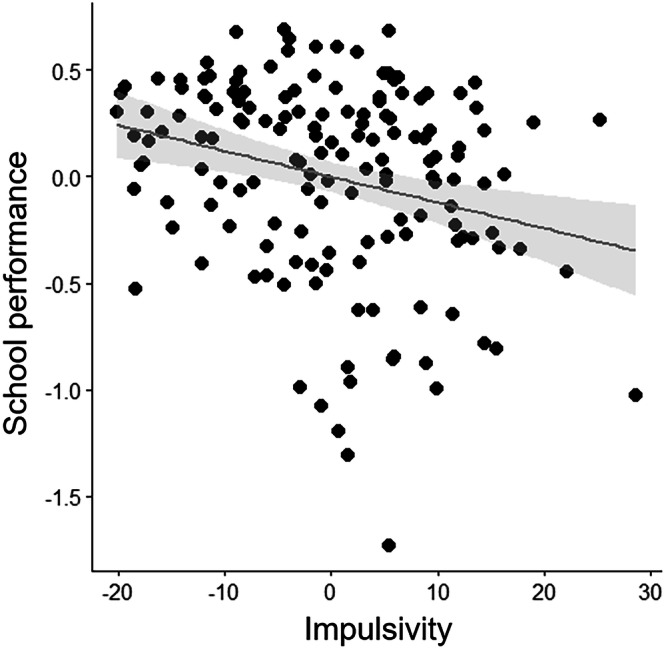
Association between impulsivity and school performance. There was a negative correlation between impulsivity and school performance (*r*-/*p* values = −0.270/*p* < 0.001). For illustration purposes, the scatterplot was created with a linear regression line added between residuals obtained after regressing out age, sex, handedness, and duration of education.

A Spearman correlation analysis was conducted between BIS-11 (impulsivity) and GPA (school performance) scores, controlling for covariates including age, sex, education, and handedness. The result showed that impulsivity was negatively associated with school performance (*r* = −0.270, *p* < 0.001). A scatter plot illustrating the relationship between BIS-11 and GPA is presented in Fig. 3.

### Mediation effects

**Fig. 4:**
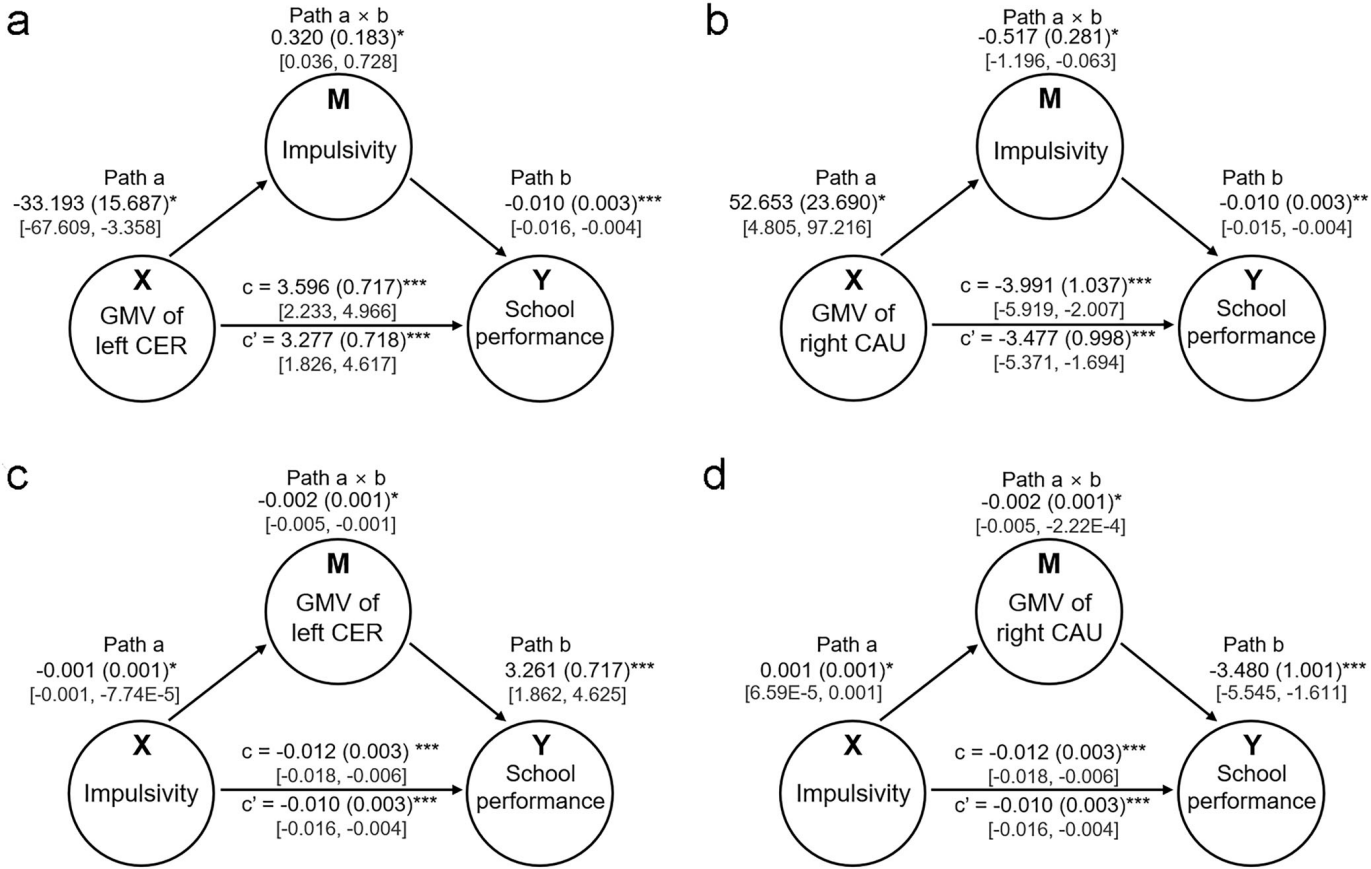
Results of the mediation analyses. **a**, **b** We investigated whether impulsivity (*M*) mediates the relationship between brain anatomy (*X*), particularly the gray matter volume (GMV) of regions identified by previous analysis in this study, and school performance (*Y*). Impulsivity was found to play a mediating role in the relationship between the GMVs of the left cerebellum **a** and right caudate nucleus **b** and school performance. **c**, **d** We further investigated whether brain anatomy (*M*) mediates the relationship between impulsivity (*X*) and school performance (*Y*). GMV of left cerebellum **c** and right caudate nucleus **d**, respectively, played a mediating role in the relationship between impulsivity and school performance. For each path, values including path coefficients, standard errors (SE), and 95% confidence intervals (CIs) are provided. Path coefficients are presented as “path coefficient (SE),” while 95% CIs are shown in square brackets “[lower, upper]”. Asterisks indicate significance levels: *p < 0.05, **p < 0.01, ***p < 0.001.

Finally, we performed a mediation analysis to determine whether impulsivity (*M*) mediated the relationship between the GMVs of the identified regions (*X*) and school performance (*Y*). After including impulsivity as an intermediate variable, the relationship between GMVs in the left cerebellum (right CN) and school performance was found to be reduced, although this effect was still significant (Figs. 4a, b). In other words, impulsivity partly mediated the relationship between GMVs of left cerebellum (right CN) and school performance. In particular, the GMV of the left cerebellum showed a positive mediation effect (path a × b), resulting from the impulsivity-associated reduction in GMV of this region (negative path a) and the negative impulsivity-school performance relationship (negative path b) (Fig. 4a). The GMV of the right CN further showed a negative mediation effect (path a × b), resulting from the impulsivity-associated increase in GMV of right CN (path a) and a negative impulsivity-school performance (path b) (Fig. 4b).

The results of the additional exploratory mediation analyses revealed that the GMVs of the identified regions (left cerebellum and right CN) played a mediating role in the relationship between impulsivity and school performance (Fig. 4c, d). In particular, the GMVs of the identified regions showed a negative, partial mediation effect on the relationship between impulsivity and school performance (please refer to Fig. 4c, d for detailed results).

## Discussion

Overall, this study is the first to examine how GMVs are linked to college students’ school performance and how these associations are mediated by impulsivity. Our results indicated that certain brain regions are associated with school performance. GMVs in the bilateral CN and left lateral orbitofrontal cortex were found to be negatively correlated with GPA, whereas GMVs in the cerebellum were positively correlated with GPA. Mediation analyses further revealed that impulsivity mediates the relationship between the gray matter structure in the brain and GPA. Further exploratory analyses confirmed that the brain gray matter structure also mediates the link between impulsivity and school performance. These findings suggest an important role of certain brain regions in school performance and further suggest that targeting impulsivity could be a key strategy to improve academic outcomes by addressing the underlying neural mechanisms.

In this study, school performance, as measured by the GPA, was found to be associated with GMV in both the CN and cerebellum. Previous research has also indicated that professional Baduk (Go game) players exhibit increased GMV in the CN^52^, while Wan et al. demonstrated that the intuition of board game experts is closely linked to the functioning of the CN^53,54^. Considering that strategy is crucial in both Baduk and other board games related to the CN, we speculated that the CN may play a key role in strategizing during academic tasks. Effective school performance requires more than just diligent efforts; indeed, it also necessitates strategic planning^55^. Students must devise strategies to select suitable courses and effective study methods to achieve or maintain high grades. Enrolling in challenging or uninteresting courses can lead to poor performance. This aligns with the findings of Iaria et al., who showed increased CN activity in individuals employing non-spatial strategies during navigation tasks^56^. Taken together, these results indicate that the involvement of the CN in strategy development is likely to affect school performance. The contradiction between our findings—a negative correlation between caudate GMV and academic performance—and a previous study of Baduk players, which reported increased GMV in the caudate nucleus among experts^52^, may be explained by differences in the cognitive demands and reward structures of the two activities. Baduk expertise requires pattern recognition, strategic planning, and the development of highly specialized cognitive skills through dedicated practice with immediate feedback^57^. In contrast, academic success in diverse college courses demands broader cognitive flexibility, self-regulation, and the ability to balance competing priorities with delayed feedback. The difference may stem from the nature of reward processing in these contexts. For Baduk players, the caudate’s role in reinforcement learning helps strengthen successful strategies through repeated practice. This may lead to adaptive enlargement of caudate structures that support expert performance. In academic settings, however, a larger caudate may reflect heightened sensitivity to immediate rewards, making students more susceptible to distractions and less able to defer gratification for long-term academic goals. This distinction highlights the context-specific role of neural structures in different cognitive domains. In contrast, individuals with larger cerebellar volumes may exhibit better school performance due to their higher cognitive function. Traditionally associated with motor control and coordination, the cerebellum has increasingly been linked to cognitive functions such as problem-solving, working memory, and language^14^. Previous research supports this finding, indicating that extensive connections between the cerebellum and various regions of the cerebral cortex are crucial for executive function^10,58^. The CN influences school performance through its role in reward-based learning and motivation, modulating decision-making processes critical for maintaining academic effort over time^9,59^. In contrast, the cerebellum supports executive functions like working memory and attention allocation through its extensive connections with prefrontal regions^14,16,60–62^. These distinct pathways highlight how structural differences in these regions contribute to academic outcomes.

Overall, our results revealed an association between several brain structures (the CN and cerebellum) and impulsivity. Previous studies have also reported that both brain structures are implicated in impulsivity, although they contribute in different ways. Research has further indicated that cerebellar dysfunction can lead to difficulties in moderating impulsive behaviors, potentially because of its role in refining cognitive and motor responses^15,16^. In contrast, the CN plays a primary role in goal-directed behavior and decision-making^63,64^. Although the head of the caudate (including the nucleus accumbens) is associated with reinforcement learning, research consistently implicates the caudate in goal-directed processes rather than habitual ones, with the latter being more associated with the putamen^65,66^. The caudate supports the acquisition and maintenance of goal-directed actions by evaluating action-outcome contingencies and integrating reward information with behavioral choices^67,68^. This role in goal-directed control is particularly relevant for academic performance, where strategic planning and flexible adjustment of behavior based on outcomes (e.g., grades, feedback) are essential. Increased CN activity or volume has also been associated with heightened impulsivity, as it can influence reward processing and drive immediate gratification^69,70^. Thus, while the influence of the cerebellum on impulsivity relates to its role in regulating responses and coordinating behavior, the role of the CN is more directly tied to the mechanisms of reward and decision-making, which affect impulsive tendencies differently. Our results reflect these previous findings, showing that individuals with larger cerebellar volumes have lower impulsivity, whereas those with larger CN volumes exhibit higher impulsivity. These opposing relationships likely reflect distinct neural mechanisms. For the CN, increased GMV has been associated with altered dopaminergic signaling, which modulates reward sensitivity and response inhibition^48,69^. Larger caudate volumes may reflect heightened reward-seeking behavior and reduced inhibitory control^70^. In contrast, larger cerebellar GMV is thought to enhance impulse control through GABAergic Purkinje cells that mediate behavioral refinement and error correction within executive control networks^16,71^.

The results of our mediation analysis demonstrated that the CN and cerebellum play crucial roles in influencing school performance, with impulsivity serving as a key mediator in this relationship. Both the cerebellum, which is involved in cognitive processing and motor coordination, and the CN, which is associated with reward processing and habit formation, contribute to impulsive tendencies. Structural variations in these brain regions can affect a student’s ability to regulate their behavior and make strategic decisions. Increased impulsivity can further impair school performance by hindering time management, adherence to study plans, and task completion^72^. This suggests that these brain structures may indirectly influence school performance by affecting the students’ capacity for self-regulation and strategic planning. Significantly, our mediation analysis demonstrated these pathways, indicating that structural variations in the CN and cerebellum do not only correlate with GPA, but rather exert a portion of their influence via their impact on impulsivity. For instance, a larger CN volume is associated with higher impulsivity, which in turn predicts lower GPA, likely by hindering the sustained focus and strategic planning necessary for academic success. Similarly, a larger cerebellum is linked to lower impulsivity, which facilitates better GPA by supporting self-regulation and cognitive control. Previously, Ivanov et al. reported that treating youths with attention deficit/hyperactivity disorder with stimulants was associated with an increased regional volume of the cerebellum^73^, and Mas-Cuesta et al. found that mindfulness training reduced CN volume, which is linked to decreased positive urgency^74^. Combined with our findings, these findings indicate that targeting the CN and cerebellum through interventions aimed at reducing impulsivity, such as therapy and medication, could enhance students’ self-regulation and strategic planning abilities and potentially improve their school performance.

Our findings extend beyond establishing a link between brain structure and academic performance, offering several practical applications. First, understanding the neurobiological basis of academic performance can inform the development of targeted cognitive training programs focused on the specific functions associated with the caudate nucleus and cerebellum. For example, interventions targeting executive functions such as planning, working memory, and inhibitory control—which are associated with cerebellar function—may be particularly effective for improving academic outcomes^14–16^. Second, recognizing that impulsivity mediates these brain-behavior relationships provides a more accessible target for intervention. Educational strategies such as mindfulness training, which has been shown to reduce caudate volume and associated impulsivity, could be implemented in college settings^74^. Similarly, structured learning environments that reduce impulsive decision-making and promote strategic planning may help students overcome neurobiological predispositions toward impulsivity^33,34,72^. Third, these findings suggest that personalized educational approaches based on individual differences in cognitive control might be more effective than one-size-fits-all interventions. By focusing on the mechanisms linking brain structure to academic performance, rather than the structural differences themselves, educators can develop more effective and targeted interventions that acknowledge the neurobiological underpinnings of learning and achievement^7,38^. However, it is crucial to emphasize that these are currently potential applications derived from correlational findings. Rigorous interventional studies are required to validate whether these approaches translate into tangible improvements in academic performance.

Overall, our post-hoc analysis revealed that impulsivity significantly impacts school performance, with both the CN and cerebellum playing key mediating roles. Impulsivity can create challenges in time management, completing assignments, and maintaining focus, all of which are crucial for academic success^33^. Dysfunction of the cerebellum, which is involved in cognitive processes, such as attention and impulse control, can impair students’ ability to regulate impulsive behaviors, leading to inconsistent school performance. Conversely, the CN, which plays a role in reward processing and habit formation, can influence impulsivity by affecting students’ tendency to favor immediate gratification over long-term goals. Increased impulsivity linked to a greater CN volume may also result in a preference for short-term rewards, potentially undermining academic effort and planning. Consequently, the interplay between impulsivity and the cerebellum and CN can significantly affect a student’s school performance by impacting their ability to regulate behavior and make strategic decisions.

Although we did not have specific hypotheses regarding hemispheric lateralization, the laterality observed in our findings—particularly the associations between the right CN, left cerebellum, and academic performance—warrants further discussion within the context of hemispheric specialization. The right caudate’s positive correlation with impulsivity may reflect its preferential role in reward processing and inhibitory control. Research has demonstrated that right striatal structures show greater activation during reward anticipation and are particularly sensitive to immediate reward cues, which could explain why right CN volume specifically relates to impulsivity tendencies affecting academic performance^75,76^. For the left cerebellum, its negative correlation with impulsivity aligns with established cerebro-cerebellar anatomical organization, where cerebellar hemispheres predominantly connect with contralateral cortical regions. The left cerebellum has been shown to connect primarily with right-hemispheric cortical regions involved in executive functions, spatial processing, and attention^77,78^. Additionally, the left posterior cerebellum has been specifically implicated in language processing and verbal working memory—cognitive functions essential for academic success across diverse college courses^79^. These lateralized connections may explain why our associations were stronger for the left cerebellum and right caudate, though future research using functional connectivity analyses could further elucidate these hemispheric differences.

This study has notable strengths and limitations in exploring the neurobiological mechanisms underlying school performance and the mediating effects of impulsivity. One key strength is that this study is the first to report how the CN and the cerebellum are associated with school performance, showing that impulsivity mediates these associations specifically in college students, diverging from previous studies focused on children and adolescents. However, this study had some limitations. First, the sample consisted solely of young adults, which may limit the generalizability of the results to other age groups. Second, the cross-sectional design of the study did not allow for causal inferences regarding the relationship between brain changes and GPA. It is important to acknowledge that while our mediation analyses suggest potential pathways through which brain structures and impulsivity might influence academic performance, the cross-sectional nature of our data prevents us from establishing true causal relationships or temporal precedence. Mediation analyses in cross-sectional data provide correlational evidence of indirect effects but cannot definitively confirm causal mechanisms. Consequently, while we discuss potential educational implications, these should be interpreted as avenues for future research rather than proven interventions, given the correlational nature of our data. Longitudinal designs would be necessary to establish whether changes in brain structure precede changes in impulsivity and subsequent academic performance, or whether academic experiences themselves influence brain development and impulsivity over time. Third, it is important to acknowledge that our study focused on structural brain differences without directly examining functional differences. We recognize that our interpretation assumes structural differences in the caudate nucleus and cerebellum translate to functional differences in self-regulation and decision-making. However, this relationship between structure and function is complex and not always direct. Structural changes in brain MRI studies are typically accompanied by functional changes; however, functional changes do not always correspond directly to structural alterations. Previous studies suggest that functional impairments often precede structural changes, gradually leading to morphological adaptations. For example, during the process of long-term potentiation (LTP) at synapses, functional modifications occur first, followed by structural remodeling of neurons within the affected synaptic region^80–83^. Future studies combining structural and functional neuroimaging approaches would help clarify how structural differences in the caudate nucleus and cerebellum functionally manifest in cognitive processes relevant to academic performance. Finally, although the study controlled for various confounding factors, such as age, sex, handedness, and duration of education, it did not account for other potential confounding variables, such as nutritional status, socioeconomic status, or intelligence quotient^84^, due to a lack of relevant data. However, information on education duration was available and used as a covariate in our analyses. While IQ and cognitive abilities may be related to GPA and impulsivity, research suggests that possessing a high IQ or specific cognitive skills does not necessarily lead to higher academic performance^85,86^. Our primary objective was to examine the influence of impulsivity on GPA. We hypothesized that lower impulsivity would help individuals maintain focus and avoid distractions during goal-directed behavior, thereby positively affecting GPA. Future research should incorporate these additional variables to clarify their potential influence on observed relationships.

This study is the first to explore the relationship between brain structures, GMV, and college students’ GPA, and how these links are mediated by impulsivity. We further found that the GMVs in the bilateral CN, left lateral orbitofrontal cortex, and cerebellum were associated with GPA. Mediation analyses also showed that impulsivity mediated the relationship between brain structures and GPA. These findings highlight the potential of targeting impulsivity to enhance school performance by addressing the underlying neural mechanisms. In order to promote academic success and personal growth and address structural inequalities in education, further research is needed to uncover how various psychological factors mediate the link between neural markers and school performance.

## Methods

### Participants

The participants were enrolled as part of a study on the Psychological and Neural Mechanisms for Predicting Academic Achievement (PNMPAA), involving an investigation of the neural mechanisms of impulsivity and risk-taking. They performed several behavioral tasks, such as an intertemporal choice task and a risk-tolerance task, and subsequently underwent brain scanning. The scanning session included high-resolution T1-weighted anatomical magnetic resonance imaging (MRI), resting-state functional MRI, diffusion tensor imaging, and functional MRI performed during the cognitive tasks. GPA values were also obtained as indicators of an individual’s overall school performance. All participants had either normal or corrected-to-normal vision and no significant medical illnesses. To ensure that our participants represent the general population, the study participants were college students currently enrolled in four-year universities located in Seoul Metropolitan Area, Daegu, and North Gyeongsang Province. Recruitment was conducted through online announcements and bulletin boards. As the majority of colleges in Korea use a 4.5 scale, all participants’ highest GPA was 4.5. No students from graduate or professional schools, such as law school, business school, pharmacy school, veterinary school, or medical school, were recruited for the study. The present study was performed in accordance with the Declaration of Helsinki. This study was approved by the institutional review boards of Daegu University (1040621-202005-HR-004). All participants provided written informed consent before participation.

Some of the collected data in the PNMPAA were used in prior studies to investigate the neural correlates of regulatory focus, which describe human motivational orientation during goal pursuit^87^ and subjective values^88–90^. In this study, to investigate the relationship among school performance, impulsivity, and brain structures in college students, we collected data on the participants’ GPA, impulsivity scores from the BIS-11, and T1-weighed MRI data. Among the participants for whom all data required for this study were available (*n* = 156), three were excluded because of outliers (>2 standard deviations [SD] from the group mean) in GPA. As such, a total of 153 was included in the final analysis [age (mean ± SD), 22.57 ± 2.77 years; 76 males/77 females; 148 right-handedness/5 left-handedness; duration of education, 15.11 ± 1.38 years; total score of BIS-11, 48.96 ± 10.28; and GPA, 3.93 ± 0.48 (range 2.30–4.50)] (Table 1).

### The Barratt Impulsiveness Scale-11

The BIS-11 is a widely used 30-item self-reported measure of impulsiveness^91^. All items are rated on a 4-point Likert scale (1 = rarely/never to 4 = almost always/always). It included six first-order (attentional, motor, self-control, perseverance, cognitive complexity, and cognitive instability impulsiveness) and three second-order (attentional, motor, and non-planning impulsiveness) factors. In many studies, the total score across all factors has been used as an indicator of an individual’s impulsiveness^34,92–96^, while the scores for each second-order factor can be used to account for individual contributions^97,98^. In this study, we focused on the total BIS-11 score to provide a comprehensive measure of general impulsivity. This approach aligns with prior research and simplifies the analysis by reducing the need for multiple comparisons across subcomponents^99,100^. Since the primary aim of our study was to examine the mediation effects of impulsivity, using the total BIS-11 score allowed us to capture the overall influence of impulsivity as a single, robust variable in the relationship between brain structure and GPA. A higher total BIS-11 score indicates a higher level of impulsiveness.

### Image acquisition and preprocessing

All imaging data were acquired using a 3 T Trio MRI scanner (Siemens, Erlangen, Germany). High-resolution T1-weighted anatomical images were obtained using a 3D magnetization-prepared rapid-gradient echo (MPRAGE) sequence [repetition time (TR) = 1,900 ms, echo time (TE) = 2.52 ms, flip angle (FA) = 9°, voxel size = 1.0 × 1.0 × 1.0 mm^3^, 192 sagittal slices].

Image preprocessing was conducted using the Computational Anatomy Toolbox (CAT), implemented in SPM12 for VBM analysis (http://www.fil.ion.ucl.ac.uk/spm/), with the default options. In brief, all T1-weighted images were corrected for bias-field inhomogeneities, after which they were segmented into the gray matter, white matter, and cerebrospinal fluid (CSF). Next, segmented images were normalized using the Shooting algorithm^101^, and resampled to 1.5 × 1.5 × 1.5 mm^3^. Jacobian determinants were applied to modulate the gray matter values in each voxel to maintain consistency. The total intracranial volume (TIV) was estimated as the sum of the volumes of the total gray matter, white matter, and CSF, as estimated from the CAT. To check the date quality, we visually inspected the preprocessed imaging data and further applied the Data Quality algorithm for VBM data homogeneity implemented in the CAT. Finally, data were smoothed with a 6 mm full-width at half-maximum (FWHM) Gaussian kernel. This decision was based on a previous study that reported subcortical regions would benefit from a smaller kernel^102^.

### Statistical analysis

All statistical analyses were conducted using MATLAB 2022a (MathWorks, Natick, MA, USA). We performed a whole-brain voxel-level regression analysis to explore the neuroanatomical correlates (the resulting GMV images) of school performance (GPA). In the regression model, we also controlled following variables: as age, sex, handedness, duration of education, and global GMV (using the global normalization option in SPM12). To exclude edge effects between gray matter and white matter, we further applied an absolute threshold of 0.1 in this analysis. Significant areas were determined using multiple comparison correction with the following settings: family-wise error rate (FWE)-corrected *p* < 0.05 at the cluster level combined with uncorrected *p* < 0.001 at the height level. Significant clusters were identified using a non-stationary cluster extent correction, which is based on random field theory described in previous studies^103,104^. This method was implemented using the NS toolbox (http://fmri.wfubmc.edu/cms/software#NS) within SPM12. This correction enhances sensitivity to spatially extended signals while maintaining statistical validity, particularly under conditions of spatially varying smoothness commonly observed in VBM data^104^.

To determine whether the regions identified from the above-mentioned whole-brain regression analysis were also associated with impulsivity, we performed Spearman correlation analyses between the GMVs of the identified regions and impulsivity (*p* < 0.05). Before performing these correlation analyses, we first calculated the residual values after regressing out age, sex, handedness, duration of education, and TIV as covariates of no interest to eliminate these confounding effects. Residual values were then used for subsequent analyses.

### Mediation analysis

The following two associations were observed in the above-mentioned analyses: the relationship between GMVs of specific regions and school performance, and between GMVs of specific regions (particularly, two clusters including the left cerebellum and right CN; see Results below) and impulsivity. Therefore, using the Mediation Toolbox (https://github.com/canlab/MediationToolbox), we performed mediation analyses to investigate whether impulsivity (*M*) could explain the effects of brain anatomy (*X*) on school performance (*Y*). In other words, in the mediation model, the dependent variable was school performance, the independent variable was the GMV of the two identified areas showing significant associations with school performance and impulsivity (see Results section), while impulsivity was set as the mediator variable. For these mediation analyses, we utilized the residual values mentioned above, along with age, sex, handedness, education duration, and TIV, to account for potential confounding effects in the statistical results. To determine the significance of the indirect effect [a × b = total effect (path c) minus direct effect (path c’)], we employed a bootstrapping procedure with 10,000 samples to generate 95% confidence intervals.

Based on the results of the above mediation analyses, we performed additional exploratory mediation analyses by shifting brain anatomy (*X*) and impulsivity (*M*). In other words, we investigated whether the GMVs of the identified regions (*M*) mediated the relationship between impulsivity (*X*) and school performance (*Y*).

## Data Availability

The data associated with this study were raw neuroimaging files that have not been made publicly available. Material transfer agreements between the research institutions are required to access these data.

## Code availability

All statistical analyses were conducted using MATLAB 2022a (MathWorks, Natick, MA, USA). No new code was generated for this study. All analyses were performed using standard, publicly available code.
